# Pediatric Supracondylar Humerus Fracture: When Should We Surgically Treat? A Case-Series

**DOI:** 10.3390/jcm14010237

**Published:** 2025-01-03

**Authors:** Filippo Familiari, Andrea Zappia, Giorgio Gasparini, Michele Mercurio, Giuseppe Tedesco, Daria Anna Riccelli, Livio Perticone, Giovanni Carlisi, Gianluca Testa, Ludovico Lucenti, Vito Pavone, Andrea Vescio

**Affiliations:** 1 Department of Orthopedics, Magna Graecia University of Catanzaro, 88100 Catanzaro, Italy; filippofamiliari@unicz.it (F.F.); dr.giovannicarlisi@gmail.com (G.C.); 2 Orthopedic and Traumatology Unit, Arnaldo Pugliese Hospital, Azienda Ospedaliero-Universitaria “Renato Dulbecco” di Catanzaro, Viale Pio X, 88100 Catanzaro, Italy; 04tedesco@gmail.com (G.T.); palanchina85@hotmail.it (D.A.R.); lperticone@icloud.com (L.P.); andreavescio88@gmail.com (A.V.); 3 Department of General Surgery and Medical Surgical Specialties, Section of Orthopedics and Traumatology, A.O.U. Policlinico Rodolico-San Marco, University of Catania, Via Santa Sofia 78, 95123 Catania, Italy; gianluca.testa@unict.it (G.T.); vpavone@unict.it (V.P.); 4 Department of Orthopaedics and Traumatology, University of Palermo, 90133 Palermo, Italy; ludovico.lucenti@gmail.com

**Keywords:** supracondylar humerus fractures (SCHFs), pediatric orthopedics, surgical timing, fracture reduction, radiological outcomes, Baumann’s angle, shaft-condylar angle, surgery postpone, after-hours surgery

## Abstract

**Background/Objectives**: Supracondylar humerus fractures (SCHFs) are the most common pediatric elbow injuries and often require surgical intervention. Despite guidelines, optimal timing for surgical management, particularly for cases without neurovascular compromise, remains unclear. This study evaluates the influence of surgical timing on short-term outcomes, focusing on fracture reduction quality and surgical parameters. **Methods**: In total, 62 pediatric patients who had been treated for Gartland type II and III SCHF between 2018 and 2023 were retrospectively assessed. Patients were grouped based on time of admission (morning, afternoon, early evening, and night shifts) and time to surgery (<12 h vs. >12 h). Primary outcomes included immediate radiological reduction, assessed via the Baumann’s angle (BA) and shaft-condylar angle (SCA). Secondary outcomes encompassed surgery duration and radiation exposure. Statistical analyses used ANOVA and chi-square tests, with *p* < 0.05 considered significant. **Results**: No significant differences were observed in BA (*p* = 0.84) or SCA (*p* = 0.79) between early and delayed surgical groups. Similarly, surgical timing (shift or delay >12 h) did not significantly affect surgery duration (*p* = 0.92) or radiation exposure (*p* = 0.12). The complication rate was 6.45%. **Conclusions**: Surgical timing, including delays beyond 12 h, does not adversely affect short-term outcomes in SCHFs. However, after-hours procedures may pose practical challenges, emphasizing the importance of surgeon experience and institutional protocols. Larger prospective studies are warranted to validate these findings and examine them in the long term.

## 1. Introduction

Supracondylar humerus fractures (SCHFs) are the most common skeletal injuries affecting the elbow in children, accounting for approximately 50% of pediatric elbow injuries [1,2,3,4,5,6]. They are also the leading indication for surgical intervention in the pediatric population [1,7,8,9].

Despite numerous published studies and clinical guidelines, SCHFs remain a subject of ongoing debate in pediatric orthopedics, with clinical decision-making often relying on the surgeon’s experience [1,4,7,10]. Gartland type II and type III fractures require surgical management, while emergent indications for surgery include a pulseless, poorly perfused arm, nerve injury, and open fractures [11].

In 2012, the American Academy of Orthopaedic Surgeons issued recommendations regarding the management of pediatric SCHFs [12]. However, the authors did not provide definitive evidence for or against a specific surgical time threshold for cases without associated neurovascular injury. A 2022 survey by the European Pediatric Orthopaedic Society revealed that two-thirds of pediatric orthopedic surgeons considered the timing of surgery a “crucial” or “important” factor influencing the outcome of SCHFs [1]. However, no conclusive evidence has emerged regarding the optimal timing for treatment of fractures without nerve or vascular complications (6, 12, or 24 h post admission). The literature does not demonstrate significant differences in postoperative complications—such as infections, neurological/vascular lesions, or compartment syndrome—based on the timing of surgery [13,14,15,16,17,18]. Notably, a higher rate of open reduction has been reported when surgery is delayed beyond 12 h, likely due to increased swelling, which complicates bloodless reduction [16].

It has been shown that orthopedic trauma surgeries performed outside of regular working hours are associated with higher complication and mortality rates. Primary contributing factors include surgeon fatigue and the absence of a specialized surgeon [17,19]. Similarly, other studies have reported that most complications in SCHF cases occur when surgery is performed outside normal daytime hours, particularly when procedures are conducted by trainees or non-pediatric orthopedic surgeons [13].

In fact, a “wait-and-see” approach is often recommended for cases occurring overnight, especially when the medical staff is not adequately experienced, as reduction and/or fixation errors are more common in surgeries performed at night, along with longer surgical durations [19,20]. According to the European Pediatric Orthopaedic Society survey, 79.3% of pediatric orthopedic surgeons suggested postponing surgery until the following morning if the patient presented after midnight, with one-quarter of respondents preferring not to perform surgery during the night shift [1]. Recent studies have also highlighted concerns related to nerve injury, as recovery times remain uncertain, and the influence of surgical timing on nerve recovery is not well understood [21]. When surgery is performed within the first 24 h, there is no consensus regarding a specific time interval that leads to better outcomes [22].

The aim of our study is to investigate how surgical timing, including the decision to delay surgery until the following morning, influence short-term outcomes in pediatric SCHF cases. We hypothesize that these factors may influence both postoperative and intraoperative variables.

## 2. Materials and Methods

### 2.1. Sample

A retrospective review of medical records from the Azienda Ospedaliero-Universitaria “Renato Dulbecco” was conducted for the period between January 2018 and September 2023. This study included patients who met the following inclusion criteria: closed Gartland type II and III supracondylar fractures, patients under 14 years of age, and complete radiological follow-up. Exclusion criteria included patients older than 14 years, those with pathological or open fractures, individuals with vascular or neurological injuries, and cases with incomplete or missing data.

### 2.2. Participants

Demographic, clinical, and radiological data were collected for each subject from the time of emergency room admission through to discharge. This included age at the time of trauma, gender, side of injury, admission time, the presence or absence of concomitant neurovascular injury, and the Gartland classification. Additional data recorded included the surgical treatment time (time from admission to surgery), surgery duration (in minutes), and X-ray exposure (in seconds) (Table 1). Based on the admission time, the sample was divided into four cohorts: Morning Shift (08:00–14:00), Afternoon Shift (14:00–20:00), Early Evening (20:00–24:00), and Night (24:00–08:00). The sample was further stratified based on the time to surgery: less than 12 h and greater than 12 h post admission (Table 1).

### 2.3. Participant Demographics

Inclusion criteria for this study were met by 62 patients. The demographic and clinical features of the participants are delineated in Table 1. The mean age of the patients at the time of trauma was 6.0 ± 2.1 years, and the male to female ratio was 1.1. The majority of the fractures were on the left side. The cohort was divided according to the time of admission into four groups according to the time of the day:

(a) Morning Shift (08:00–14:00): 13 patients, mean age 6.1 ± 2.4 years;
(b) Afternoon Shift (14:00–20:00): 20 patients, mean age 6.6 ± 2.5 years;
(c) Early Evening (20:00–24:00): 11 patients, mean age 5.3 ± 2.8 years;
(d) Night Shift (24:00–08:00): 18 patients, mean age 6.3 ± 2.4 years.

Additionally, patients were divided into two subgroups based on time from hospital admission to surgical treatment:

I. Surgery in 12 h: 37 patients, mean age 6.6 ± 2.9 years;
II. Surgery after 12 h: 25 patients, mean age 5.5 ± 1.7 years.

There was no significant difference between the groups in terms of gender, age, and the side involved (*p* > 0.05). In four cases (6.45%), acceptable fracture reduction could not be achieved.

### 2.4. Surgical Intervention

Percutaneous pin fixation, followed by closed reduction, was performed within 72 h of emergency department admission. General anesthesia was administered, and the patient was positioned in the prone position, with the affected arm placed on the C-arm fluoroscopy plate. True lateral and anteroposterior (AP) fluoroscopic views were exclusively employed to ensure precise fracture reduction. Fixation was achieved using 1.6 to 2 mm Kirschner wires (K-wires), configured in either a lateral or crossing-pin arrangement. 

The choice of pin configuration was influenced by factors such as elbow swelling, the type of fracture, and the surgeon’s expertise and preference.

For the crossing-pin configuration, the first wire was inserted centrally through the lateral condyle, while the second was positioned medially, targeting the anterior aspect of the medial epicondyle while avoiding the ulnar nerve.

In the lateral configuration, the first wire was aligned parallel to the olecranon, with additional wires inserted laterally or medially as needed to correct any residual fracture instability. Post-fixation stability was verified fluoroscopically by confirming alignment and the absence of displacement during simulated joint movements. Postoperatively, patients were immobilized with a posterior splint at 90° elbow flexion to optimize circulation and reduce swelling. The protruding K-wires were left exposed to facilitate removal. Most patients were discharged within 24 h and underwent weekly clinical follow-ups during the first month. At the four-week follow-up, an elbow radiograph was obtained to confirm fracture healing, and the K-wires were removed. Subsequent clinical evaluations were conducted at one, three, six, and twelve months after surgery, with annual assessments continuing until skeletal maturity.

Rehabilitation to restore elbow range of motion commenced immediately after K-wire removal. Functional follow-ups monitored the recovery of elbow mobility and overall joint functionality. Radiological assessments focused on verifying fracture alignment and healing progression. Patients were cleared to resume sports and full physical activity three months post trauma, contingent upon satisfactory clinical and radiological outcomes.

### 2.5. Primary Outcome

The primary outcome of this study was the immediate radiological reduction of the fracture. The posteroanterior view was assessed using Baumann’s angle (BA), defined as the angle between the humeral axis and a line through the epiphyseal plate of the capitellum. The normal range for BA is 64–81 degrees, with a mean value of 72 degrees (commonly cited range: 75–80 degrees) [23]. In the lateral view, the shaft-condylar angle (SCA) was used as the assessment parameter. The SCA is formed by the axis of the humerus and the capitellum in the lateral view, with a normal value of <40° [24]. Radiological measurements were performed by two authors (G.C. and A.V.).

### 2.6. Secondary Outcomes

Secondary outcomes included radiation exposure (measured in seconds) and surgery duration (measured in minutes).

### 2.7. Statistical Analysis

Continuous data are presented as mean values and standard deviations. Analysis of variance (ANOVA) was used to compare demographic characteristics (excluding gender and side), as well as primary and secondary outcomes (excluding complications). Chi-square tests were used to assess the homogeneity of the cohorts with respect to gender and side of injury. A *p*-value of <0.05 was considered statistically significant. All statistical analyses were performed using GraphPad Software (2016 version, GraphPad Inc., La Jolla, CA, USA).

## 3. Results

### 3.1. Primary Outcome: Immediate Radiological Reduction

The mean BA in the posteroanterior view was 76.8 ± 12.8 degrees. In the lateral view, the mean SCA was 34.5 ± 10.9 degrees. A comparison of radiological outcomes between cohorts (surgery < 12 h vs. >12 h) showed no significant difference in the BA (*p* = 0.84) and SCA (*p* = 0.79) between the groups. After stratification of the cohort according to BA and SCA (morning, afternoon, early evening, night), no significant differences were found in the primary outcome (*p* = 0.16 and *p* = 0.08, respectively) (Table 2).

### 3.2. Secondary Outcomes

Radiation Exposure: The average time of radiation exposure for all patients in whom the surgical intervention was performed was 96.0 ± 67.1 s. There were no significant differences in radiation exposure between patients who received surgical intervention within 12 h from admission compared to those whose surgery took place after this time frame (*p* = 0.12). No significant difference in radiation exposure was noted across all time cohorts (morning, afternoon, early evening, night) (*p* = 0.77) (Table 3).

Duration of Surgery: The mean duration of surgery was 34.4 ± 20.4 min. No significant differences in duration of surgery were found between the patients who underwent surgical management within 12 h of admission and the patients who had undergone surgery after 12 h (*p* = 0.92). Statistically significant differences in the secondary outcomes, including radiological reduction and surgical duration, were found when analyzed according to the timing of the surgical procedure: morning, afternoon, early evening, night. Indeed, no trend suggesting either a decrease or an increase in surgical times across the cohorts could be identified (*p* = 0.38) (Table 3).

## 4. Discussion

The aim of the current study was to evaluate the influence of surgical timing on the SCHF outcomes with particular attention to the quality of immediate reduction and intraoperatory parameters. The primary outcome showed no differences in terms of fracture reduction when treated in evening and night hours or after 12 h from ED admission. As for the secondary endpoints, there were no significant differences between groups with respect to the duration of the surgical procedure and amount of radiation exposure, even after fractures had been treated during the evening and nighttime hours. The current results are consistent with previous research that reports that delays in surgical timing were not significantly associated with worse clinical outcomes but did show the potential disadvantages of after-hours surgery: longer operative time and more radiation exposure. For instance, Shon et al. [25] stratified their cohort by time to surgery (i.e., <6 h, 6–12 h, and 12–24 h from injury) and found no difference in reduction quality or loss of reduction based on timing. Likewise, other studies by Schmid et al. [26] and Iyengar et al. [27] failed to show any significant difference in complication rates or surgical outcomes that were time to treatment related and thus supported the hypothesis that delayed reduction is not associated with poor clinical results where proper management of the fracture was performed. Although there was some deviation in the surgical time and radiation exposure, for the secondary outcome of complications, we did not find any significant difference between fractures managed during daylight hours and those managed in the evening or night and when operative treatment was prolonged beyond 12 h. Our complication rate of above 6% corresponds with the results from studies in the literature. Paci et al. [19] found that fractures treated outside regular hours were more likely to be severe, particularly those treated in the late-night hours, but complication rates (including malunion) were similar regardless of timing. Importantly, the present study included the assessment of immediate reduction according to BA and SCA normal values, an analysis that has not always been considered in previous studies [28]. Such consideration further underlines the fact that delaying surgical treatment does not appear to adversely affect fracture healing in any significant way. On the contrary, Paci et al. [19] found a higher rate of malunion in fractures treated overnight (23:00–06:00). This result is perhaps explained by confounding variables, so that fractures treated at after-hours had worse characteristics and there was a higher possibility that these fractures were treated by less-experienced surgical staff such as fellows and residents. 

The present study did not find such a correlation, which may reflect the more controlled setting of surgical operations in our hospital, where even operations performed outside regular hours are carried out by experienced surgeons or under supervision. An important finding of this study was that there was no increase in operating time and no increased exposure to radiation in fractures treated during evening and night shifts. In contrast, Terpstra et al. [29] showed that surgeries performed after regular hours are generally longer, likely due to factors such as inexperienced personnel and the lack of availability of key resources. Previous findings are supported by the data reported by Tuomilehto et al. [20], who observed a reduction in surgery duration by an average of 11 min. The study also documented a decrease in operations lasting over 60 min and a reduction in the number of procedures exceeding 120 min. Similarly, Aydoğmuş et al. [30] reported an 8 min reduction in surgery duration. Furthermore, the authors highlighted a higher dissatisfaction rate associated with surgeries performed during night shifts, reported by 43% of orthopedic surgeons, 81% of anesthesiologists, and 84% of nurses [14]. The duration of surgeries is also linked to the mean cost of operating room time, which, based on the literature, is estimated at USD 46.04 per minute [31]. Notably, reductions in on-call operating room costs are associated with improved staff satisfaction, with dissatisfaction rates among night-shift staff mirroring those previously mentioned [20]. Further, the increased exposure to fluoroscopy during nocturnal procedures is alarming, since increased radiation exposure has been associated with potential long-term health effects [15]. This might be associated with the use of less experienced surgical teams during after-hours, as seen in the study by Paci et al. [19], where non-pediatric orthopedic surgeons were more likely to perform after-hours procedures. This, in turn, leads to more time used in decision-making, positioning, and fracture reduction—altogether, the duration of surgery and, consequently, radiation exposure are prolonged. As reported in another study [9], surgical experience did not differ significantly between the groups, suggesting other causes of these outcomes, such as differences in institutional protocols and differences in surgical complexity. Aydogmus et al. explained [30] that 35% of the cohort underwent treatment during the evening or overnight hours and hypothesized that SCHFs treated during the night tended to be more severe. However, several studies [19,27] have noted that worse fractures may not necessarily result in worse outcome when treated during after-hours, in which the authors inferred that delayed treatment per se is not deleterious. Rather, the severity of the fracture and the availability of experienced surgeons are most likely more related factors in determining outcome.

One important observation in the current study is the lack of a standardized definition of “after-hours” surgery across the literature. Studies often define after-hours as any time outside regular office hours, but the exact timing varies significantly between studies. For example, Paci et al. [19] defined after-hours as between 16:00 and 06:00, while others, such as Wendling-Keim [28], specified the time frame as 22:00–02:00. This variability makes it difficult to directly compare results across studies and complicates the interpretation of findings related to the timing of surgery. Additionally, the literature shows substantial variation in the clinical and radiological parameters used to assess the SCHF outcomes. While some studies report on the Baumann’s angle and humeral shaft angle, others focus on different measures, such as the carrying angle or range of motion [10,32,33]. This variability may contribute to discrepancies in the reported outcomes and complicate the synthesis of evidence regarding optimal treatment timing. Another key point is the variation in the specialties of the surgeons performing pediatric SCHF surgery. In the present study, there is a lack of consistency in whether procedures are performed by pediatric orthopedic surgeons, general orthopedic surgeons, or surgical fellows. The findings have suggested that outcomes may vary depending on the level of expertise of the treating surgeon, which may confound the results. This variation in surgical expertise underscores the importance of standardized training and protocols to ensure optimal care for pediatric SCHFs, particularly in after-hours settings.

This study has several limitations. Firstly, it is a retrospective analysis, which inherently carries the risk of selection bias due to the observational nature of the study. Additionally, the relatively small sample size (62 patients) limits the generalizability of our findings. Future research should aim to validate these results through a prospective study design with a larger patient cohort. Another limitation is the potential for measurement bias, particularly in the assessment of radiographic outcomes. The lack of standardized protocols for measuring angles may have introduced inter-rater variability in our assessments. Furthermore, no additional analysis was performed to compare Gartland type II and type III fractures due to the limited number of cases in these subgroups, which could have resulted in overanalyzing a small sample size.

Despite these limitations, this study has several strengths. It represents a single-institution cohort with a relatively homogeneous surgical practice, which reduces variability in treatment protocols and outcomes. Additionally, our study includes a thorough analysis of both primary and secondary outcomes, such as fracture reduction and surgical times, allowing for a comprehensive evaluation of the effects of surgical timing on pediatric SCHFs.

## 5. Conclusions

In conclusion, our study demonstrates that the timing of surgery for pediatric supracondylar humerus fractures does not significantly affect the quality of fracture reduction or complication rates. However, after-hours surgery was not associated with longer operative times and increased radiation exposure but may contribute to additional risks. These findings support the notion that, while surgical delay is generally not harmful, it could be reasonable considering the practical drawbacks of after-hours procedures, particularly in terms of resource utilization and surgeon experience, according to the recent literature. Further prospective studies with larger sample sizes are needed to confirm these findings and explore the potential impact of surgical timing on long-term outcomes, including functional recovery and deformity.

## Figures and Tables

**Table 1:** Demographic and clinical characteristics of the participants.

Groups	Sample	Morning Shift	Afternoon Shift	Early Evening	Night	<12 h	>12 h
Number	62	13	20	11	18	37	25
Gender (Male/Female)	33/29	7/6	9/11	6/5	11/7	19/18	14/11
		*p* = 0.80				*p* = 0.72	
Age (years (stdev))	6.0 ± 2.1	6.1 ± 2.4	6.6 ± 2.5	5.3 ± 2.8	6.3 ± 2.4	6.6 ± 2.9	5.5 ± 1.7
		*p* = 0.58				*p* = 0.09	
Side (Right/Left)	27/35	5/8	9/11	4/7	9/9	16/21	11/14
		*p* = 0.88				*p* = 0.95	

**Table 2:** Primary outcome results.

Groups	Morning Shift	Afternoon Shift	Early Evening	Night	<12 h	>12 h
postop Baumann’s Angle	77.8 ± 7.3	74.8 ± 5.5	74.4 ± 4.7	73.2 ± 4.5	75.3 ± 5.9	75.0 ± 5.1
*p*	*p* = 0.16				*p* = 0.84	
postop Shaft-Cond. Angle	36.3 ± 6.9	35.8 ± 10.1	43.5 ± 11.2	34.7 ± 8.2	37.2 ± 9.8	37.9 ± 10.5
*p*	*p* = 0.08				*p* = 0.79	

**Table 3:** Secondary outcome results.

Groups	Morning Shift	Afternoon Shift	Early Evening	Night	<12 h	>12 h
Radiation Exposure (s)	83.2 ± 59.3	92.5 ± 81.1	106.4 ± 62.1	102.4 ± 30.6	104.1 ± 75.2	102.1 ± 74.7
*p*	*p* = 0.77				*p* = 0.92	
Surgery Duration (min)	24.3 ± 10.6	33.5 ± 25.4	41.8 ± 42.9	32.5 ± 11.8	37.2 ± 23.0	28.2 ± 20.7
*p*	*p* = 0.38				*p* = 0.12	

## Data Availability

All data are available upon reasonable request.
